# An Investigation of Risk Factors Associated with Tuberculosis Transmission in South Africa Using Logistic Regression Model

**DOI:** 10.3390/idr14040066

**Published:** 2022-08-18

**Authors:** Tshepo Frans Maja, Daniel Maposa

**Affiliations:** Department of Statistics and Operations Research, University of Limpopo, Private Bag X1106, Sovenga, Polokwane 0727, South Africa

**Keywords:** demographic, logistic regression, socio-economic and health factors, South African Demographic and Health Survey, tuberculosis

## Abstract

**Background:** South Africa has a high burden of tuberculosis (TB) disease and is currently not meeting the national and international reduction outcome targets. The TB prevalence rate of South Africa in 2015 was estimated at approximately 690 per 100,000 population per year, with an incidence rate of about 834 per 100,000 population. This study examines risk factors associated with development of TB in South Africa. **Materials and Methods:** This study utilised readily available open access secondary data of 2019 South African Health and Demographic Survey from Statistics South Africa (StatsSA) website, which was collected from self-reported information relating to TB in the household questionnaire. The factors analysed were of demographic, socio-economic and health nature. Bivariate and binary logistics analyses were carried out from which appropriate inferences were drawn on the association of TB with demographic, socio-economic and health factors. **Results:** In multivariate analysis the study revealed that age, personal weight, smoke, alcohol, asthma, province of residence, race and usually coughing were significantly associated with an increased risk of having TB. **Conclusions and Recommendations:** The results strongly suggest that young and older people coming from black and coloured ethic groups, who are asthmatic and cough frequently, and/or smoking and consuming alcohol are at high risk of developing TB. In addition, those who are overweight appear to have an increased risk of TB transmission, with the Western Cape, Eastern Cape, Northern Cape, Free State, North West and Gauteng being the hardest hit provinces. Hence, the study recommends that these factors must be taken into account in the planning and development of TB policies in order to work successfully towards the achievement of sustainable development goal of reducing TB by 80% before 2030.

## 1. Introduction

Tuberculosis (TB), a dangerous and deadly chronic infectious disease, remains one of the world’s leading public health challenge and is now considered the world’s leading infectious killer alongside human immunodeficiency virus/acquired immune deficiency syndrome (HIV/AIDS) [1,2]. In 2015, approximately 10.4 million people were infected with TB and 1.4 million people died from the disease worldwide [3]. Home to 11% of the world’s population, Africa bears 29% of the global burden of TB cases and 34% of related deaths, and the challenges of fighting TB in the continent have increased [4]. TB causes major health problems and is an enormous economic burden for the population of most low-income countries.

South Africa, located in the southern region of Africa, is considered to have one of the most severe TB epidemics in the world and ranks fifth among the 22 high-burden countries. According to [5,6], South Africa’s TB prevalence rate in 2015 was estimated to be approximately 690 per 100,000 population, per year, with an incidence rate of about 834 per 100,000 population. Although progress has been made in reducing TB infection in recent decades in South Africa, more efforts are needed to accelerate the decline. There is need for improved diagnosis and treatment of all forms of TB for the long-term control and elimination of TB. Despite all the remarkable advances in trying to reduce TB infection which is preventable, the disease remains unacceptably high and efforts to combat it need to be accelerated.

According to [7], the risk of TB infection is mainly determined by various factors. Hence, knowledge and understanding of risk factors associated with TB is crucial for developing effective prevention measures. Several studies have been performed in various countries to identify predictors of TB infection [3,5,6,8,9,10]. For instance, ref [9] investigated the association of smoking and outcome of TB, and found that the risk of developing TB was higher in smokers in comparison to non-smokers. Furthermore, ref [10] conducted a study to identify key social determinants of TB and discovered that social factors such as poor living and working conditions, HIV infection, smoking, alcohol abuse, diabetes and indoor air pollution are associated with high risk of TB transmission. Due to a rise in the prevalence rate of people with TB over the past two decades an interest in the demographic, socio-economic and health determinants of TB has grown worldwide. As a result, a review of the literature identified some risk factors for TB which include, but are not limited to, smoking, low body mass index, level of education, alcohol consumption, diabetes and drugs [7,8,11,12]. However, factors underlying the observed increase of TB in South Africa as a whole remain unclear since the majority of the studies conducted were outside the country. In South Africa, limited studies which assessed the importance of these factors were conducted in several provinces.

As South Africa aims to eliminate TB and achieve the international targets, it is important to assess the relative importance of various factors which may be interrelated using the database of the whole country in order to understand the current pattern and causes of TB. Accordingly, ref [13] indicated that TB is being diagnosed too late for effective treatment and therefore, to save lives and prevent the onwards transmission of the infection, important factors need to be identified. The study of these factors will provide a crucial opportunity to develop a reliable profile of those at high risk of developing TB infection and the findings may aid in the early identification of effective interventions to prevent the occurrence of this infection.

It was against this background that this research was conducted. The results of this research will help South Africa’s National Tuberculosis Control Program, the Ministry of Health, and the community to develop effective intervention strategies in order to prevent the onset of the disease. In addition, the results of this paper will also uncover the routines and lifestyles of people with TB and take precautionary measures to reduce the percentage of TB cases in South Africa. The aim of this paper is to identify associated socio-economic, demographic and other proximate factors which influence the occurrence of TB in South Africa.

## 2. Materials and Methods

### 2.1. Materials

#### 2.1.1. Source of Data

This study is based on secondary analysis of data from the 2019 South African Demographics and Health Survey (SADHS) dataset obtained from the Statistics South Africa (StatsSA) website. The SADHS data is readily available and accessible on a public domain website. The 2019 SADHS dataset contains a wealth of information on the individual characteristics of 7768 South African citizens. The dataset contains data on socio-economic, demographic, and health-related variables that were collected and recorded into Statistical Package for Social Sciences (SPSS) version 15.0.

#### 2.1.2. Description of Variables

A thorough study of TB among South Africans was conducted and special attention was paid to some explanatory factors that influence the likelihood of developing TB, which is the main focus of the study.

##### Dependent Variable

The outcome variable of this study is TB infection. TB refers to a chronic, lifelong infection that usually attacks the lungs. It can also spread to other parts of the body such as the brain and spinal cord. A type of bacteria called Mycobacterium TB causes it. The outcome variable, TB, was classified as successful if an individual had been diagnosed with TB or failure if an individual was not diagnosed with TB. Success is coded 1 and failure is coded 0. This means that people in South Africa who were diagnosed with TB are compared to people who were not diagnosed with TB in the study.

##### Explanatory Variables

The explanatory variables available for this study were classified as socio-economic, demographic, and health-related variables as presented in Table 1. The table also shows how the variables were recorded for the purposes of this study. There are a total of 13 variables that were used in this study as risk factors and selected for building a predictive model.

### 2.2. Method of Data Analysis

#### 2.2.1. Test of Association

The chi-squared test of independence (also known as the Pearson chi-squared test) is a nonparametric statistic, most useful for testing hypotheses when the variables are nominal, as often happens in clinical research [14]. This test consists of two variables such as groups and categories, and seeks out to determine if one variable is independent of the other. In this study, the chi-squared test is used to determine whether there is a significant association between explanatory variables in the dataset and the outcome variable TB. According to [15], unlike other nonparametric and some parametric statistics, the calculations required to obtain chi-squared test statistic provide sufficient information about how each group is performed in the study. This breadth of detail enables the researcher to understand the results and obtain more detailed information from this statistic than from others. Under this test, the null hypothesis states that there is no association between the outcome variable, TB, and each of the explanatory variables whereas the alternative hypothesis states that there is an association between the outcome variable TB and each of the explanatory variables. Furthermore, the test statistic is a chi-squared random variable $\chi^{2}$ defined by the following equation:

(1) $$\chi_{cal}^{2}=\sum_{i=1}^{r}\sum_{j=1}^{c}\frac{{\left(O_{ij}-E_{ij}\right)}^{2}}{E_{ij}}$$

where, $\chi_{cal}^{2}$ is the calculated chi-squared value, *c* and *r* represent the levels of the first and second variable, respectively. $O_{ij}$ and $E_{ij}=\frac{n_{i}n_{j}}{n}$ denote the number of observations or actual frequency and the expected frequency in a cell with *n* denoting the sample size, respectively.

Accordingly, if the findings are unlikely, the investigator rejects the null hypothesis. Generally, this involves comparing the *p*-value to the significance level, α, and therefore, null hypothesis is rejected if the *p*-value is less than α=0.05. Therefore, if null hypothesis is rejected, we conclude that there is statistical evidence or significant association between the two tested variables. However, if the null hypothesis is not rejected, we conclude that there is no statistical evidence or no significant association between the two tested variables.

#### 2.2.2. Logistic Regression

Logistic regression is a mathematical modelling approach used to determine how efficient independent variables are on the dependent variables [16]. It is usually used as a statistical model in experimental studies involving categorical dependent variables and it aims to find a model that predicts the outcome of the dependent variables. In general, the dependent variable in logistic regression is dichotomous or binary such as success or failure. In other words, for binary dependent variable, the event of interest is coded as 1 and the non-event as 0, while the independent variables are a mixture of both quantitative and qualitative variables. Therefore, in this study, the choice of this model was because the desired results are two possible outcomes of “TB status” and were coded as 0 and 1. That is, if *Y* denotes a dependent variable, then, $Y=\left\{\begin{array}{cc} 1 & if\;\;TB\;exists\;(event)\\ 0 & if\;\;TB\;does\;not\;exist\;(\mathrm{non-event}). \end{array}\right.$

The logistic regression model has the following form:

(2) $$\pi \left(x\right)=\frac{exp(\beta_{0}+\beta_{1}X_{1}+\beta_{2}X_{2}+\ldots +\beta_{k}X_{k})}{1+exp(\beta_{0}+\beta_{1}X_{1}+\beta_{2}X_{2}+\ldots +\beta_{k}X_{k})},$$

where π(x) is a conditional probability that the outcome is present, $\beta_{0},\beta_{1},\beta_{2},\ldots ,\beta_{k}$ are known as regression coefficients and $X_{1},X_{2},\ldots ,X_{k}$ are independent variables of interest. Our model will be predicting the logit, and the logistic regression function is the logit transformation of π(x), where

(3) $$w=logit\left(\pi \left(x\right)\right)=ln\frac{\pi (x)}{1-\pi (x)}=\beta_{0}+\beta_{1}X_{1}+\ldots +\beta_{k}X_{k}.$$

Therefore, using a logistic transformation in this way overcomes the problems that could arise if π(x) were directly modelled as a linear function of random variables [17]. In particular, it avoids fitting probabilities outside the range of 0 and 1.

##### Fitting of Logistic Regression Model

By fitting the model, we can estimate the logistic regression coefficient of the selected variables. Therefore, in logistic regression, the maximum likelihood estimation method is used to estimate the model coefficient. In other words, maximum likelihood estimation finds the best values for

(4) $$w=\beta_{0}+\beta_{1}X_{1}+\ldots +\beta_{k}X_{k}.$$

According to [16], the maximum likelihood estimation involves two tests, namely, the Wald test and the likelihood ratio test and these tests can be used to assess the significance of an independent variable in logistic regression. The Wald test is more like a *Z*-test, which means that its test statistic nearly have standard normal values whereas the likelihood ratio test is a chi-squared statistic that gets its benefits from maximised likelihood values.

##### Wald Test

The Wald statistics are defined as the ratio of the estimated coefficient to its standard error. In this study, it was used to test the significance of each independent variable. The Wald test has the following hypotheses:

**Hypothesis** **1** **(H1).** *$\beta_{i}=0$ (Independent variables have no significant effect on the log odds ratio).*

**Hypothesis** **2** **(H2).** *$\beta_{i}\neq 0$ (Independent variables have a significant effect on the log odds ratio).*

Wald test statistics is calculated as:

(5) $${\left(\frac{coefficient}{SE_{coefficient}}\right)}^{2}={\left(\frac{\beta_{i}}{SE_{\beta_{i}}}\right)}^{2},$$

where SE denotes the standard error. According to [18], each Wald statistic is compared with a chi-squared distribution with one degree of freedom. If the Wald statistic is significant, the *p*-value must be less than 0.05, and then the parameter is considered to be useful in the model [19].

##### Likelihood Ratio Test

The test statistic of the likelihood ratio test is calculated as:

(6) $$L=-2ln(\frac{L_{0}}{L_{1}}),$$

where $L_{0}$ represents the likelihood of obtaining the data when a parameter is zero and $L_{1}$ represents the likelihood of obtaining the data evaluated at the maximum likelihood estimation of the parameter. The statistic is compared with chi-squared distribution with one degree of freedom.

##### Odds and Odds Ratio

The odds of an event is defined as the probability of an event occurring divided by the probability of an event not occurring and it is given by

(7) $$odds=\frac{p_{i}}{1-p_{i}},$$

where $p_{i}$ represents the probability that an event (TB) will happen. Therefore, the odds ratio (OR) is simply the ratio of the two odds. According to [20], OR is commonly used as a measure of the magnitude of the detected relationship between variables. Hence, the formula for OR of the disease or an event is given by

(8) $$OR=\frac{The\;odds\;of\;the\;disease\;when\;the\;disease\;exists}{The\;odds\;of\;the\;disease\;when\;the\;disease\;does\;not\;exist}=\frac{\frac{P_{1}}{1-P_{1}}}{\frac{P_{0}}{1-P_{0}}}.$$

Since $\beta =log(\frac{\frac{P_{1}}{1-P_{1}}}{\frac{P_{0}}{1-P_{0}}})$ therefore it implies that $OR=e^{\beta }$, where β is an estimated coefficient. As a result, an OR more than one, implies that an increasing value in the variable corresponds to increasing odds of the event occurrence, meaning that the factors are more risky, whereas an OR less than one, implies that an increase in value in the variable corresponds to decreasing odds of the event’s occurrence, meaning that factors are less risky.

##### Model Building and Steps for Variable Selection

In this study, the following steps were used to build the model. First, the selection process must begin with a bivariate chi-squared test to test the relationship between each independent variable and TB. Second, the choice of independent variables for the multivariate logistic regression analysis will correspond to the results of the bivariate analysis. As a result, all variables that showed a significant relationship in the bivariate analysis were then included in the multivariate logistic regression analysis. Finally, the importance of each independent variable included in the multivariate logistic regression model should be verified by a step-by-step selection procedure. Therefore, forward step-by-step selection procedure was employed to select variables which influence jointly the dependent variable, TB.

According to [21], forward step-by-step selection procedure involves starting a model that does not include any of the explanatory variables. Therefore, all explanatory variables that are not initially in the model are examined for their *p*-values and variables with *p*-values that are less than the specified value of 0.05 are added to the model. Eventually, the explanatory variables left out of the analysis at the last step all have *p*-values larger than 0.05 and as a result no more are added. In other words, the procedure continues until no new explanatory variable can be added.

## 3. Results

### 3.1. Descriptive Statistics for Dependent and Predictor Variables

The total sample included 7768 South African citizens who are 15 years or older, with majority of them coming from Eastern Cape (EC) province (36.5%) and Northern Cape (NC) province (15.0%), respectively. The distribution of the sample was such that 57.6% were women and 42.4% were men, with at least 60% of these participants living in urban areas. The most prevalent population group was black Africans (63.5%), and with almost all age groups contributing around 20% of the participants. The source of drinking water of most participants (66.6%) was from piped water and more than half (54.0%) used flush toilet facilities with 65.0% of them had access to electricity. A large group (60.8%) of the participants had normal weight, with 40.7% and 42.2% being daily smokers and current alcohol users, respectively. A small proportion (15.4%) and (4.3%) of the participants had hypertension and asthma, respectively. A sizeable proportion (13.1%) of the participants reported that they usually cough.

### 3.2. Chi-Squared Test of Association for Risk Factors

Based on Table 2, independent variables were tested by chi-squared test to study their association with TB. There is an association between TB and race group, province of residence, age, smoke, alcohol, source of drinking water, electricity, cough, personal weight, gender and asthma since their *p*-values are less than the significance level of 0.05. This suggests that these independent variables are associated or contribute to TB in people. However, it is also important to note that there are no associations between TB and type of place of residence as well as hypertension because their *p*-values were higher than the significance level of 0.05.

### 3.3. Multivariate Analysis

The chi-squared test, described in the previous section, tests for an association between the dependent variable and each independent variable. However, it does not take into account the influence of other variables, nor does it determine the direction of the association. To address this, multivariate logistic regression was used to test the significance of the independent variables in influencing a dependent variable in the presence of other variables considered. Accordingly, to understand the variables associated with TB, only significant variables obtained from chi-squared test, were considered and tested in a multivariate logistic regression model using the forward elimination method.

Table 3 shows the results of the multivariate logistic regression analysis model of TB in South Africa. The table shows that the variables that were significant after the last step of forward elimination model were included in the final model since their *p*-values were less than 0.05. The variables, which were found to be significantly associated with TB were age, personal weight, smoke, alcohol, asthma, province of residence, usually cough and race. Source of drinking water and electricity, which were significant in the bivariate analysis, were not significant in the multivariate logistic analysis. This implies that, controlling for the effects of other variables, source of drinking water and electricity had no significant impact on TB.

Multivariate analysis showed that participants aged 15–24 years were 2050 times more likely to develop TB than participants aged 54 years and older after controlling for other variables in the model (OR = 2.050, *p*<0.001, CI = 1405, 2.990). Conversely, people between the ages of 45 and 54 years were 23.8% less likely to get TB than people over the age of 54. While odds of participants between the ages of 25–34 and 35–44 were not significant, indicating that the chance of having TB among people in these age groups is similar to that in people over 54 years and older.

Underweight and normal-weight people were 73.4% and 43.1%, respectively, less likely to develop TB as compared to those who are overweight, which is the reference group. For smokers, the odds ratio is 0.739. This implies that smoking people are 0.739 more likely to develop TB than non-smokers (OR = 0.739, *p* = 0.029, CI = 0.563, 0.969). The odds ratios for those who drink alcohol and having asthma are 0.619 and 0.580, respectively. Therefore, this implies that people who drink alcohol are 0.619 times more likely to develop TB as compared to those who do not drink alcohol (OR = 0.619, *p* < 0.001, CI = 0.475, 0.807), whereas people with asthma were 0.580 times more likely to develop TB than those without asthma (OR = 0.580, *p* = 0.015, CI = 0.374, 0.900).

In addition, people residing in the WC, EC, NC, FS, NW and GP are 5708, 6815, 7433, 12.030, 2771 and 4794 times more likely to develop TB, respectively, compared to those who reside in LP, which is the reference category. However, the study found that the odds of people living in KZN and MP were not significant, suggesting that the chance of having TB among people residing in these provinces is similar to those in LP. Additionally, the results of this study show that people who are from black/African as well as Coloured race group are 13.184 and 8855, respectively, times more likely to develop TB than those coming from Indian population group. Finally, the odds ratio of people who usually cough is 0.424, which implies that people who usually cough are 0.424 times likely to develop TB than those who do not. As observed in Table 4, demographic, socio-economic and health factors are statistically significant in influencing the risk of having TB.

### 3.4. Model Dignostics

Hosmer-Lemeshow test, was used to assess the goodness of the fitted model. According to [22], an overall goodness of fit of the model is indicated by *p*-values greater than 0.05. Therefore, since the *p*-value is 0.476 in Table 4, which is greater than 0.05, we conclude that the dataset fit the model very well.

## 4. Discussion and Conclusions

This study examined the role of certain bio-demographic, socio-economic and health-related factors as determinants of TB in South Africa using a binary logistic regression model. Binary logistic regression model is a type of regression model where the dependent variable is binary. One of the important steps in achieving a reduction or even elimination of TB by 2030 is to carefully understand the underlying determinants or predictors of TB outcome. As a result, this study investigated the determinants of TB in South Africa.

The results showed that several factors are involved in TB transmission. After the application of logistic forward elimination method to factors found to be associated with TB in a bivariate analysis, it was revealed that age, personal weight, smoke, alcohol, asthma, province of residence, race and usually coughing were significantly associated with TB. The results of our study are similar and consistent with the available literature, which points out that these factors are strong predictors of TB [3,7,23,24,25]. For instance, several studies also identified age to be significantly associated with TB [7,25]. The age factor, as expected is more strongly associated with TB in South Africa. It has been suggested that this could be attributed to the decreased immune status of the elderly, which makes them more prone to developing TB [3]. Both the multivariate and the bivariate analysis showed that smokers or alcoholics were at a higher risk of developing TB. These results are consistent with the available literature, which suggests that smoking and alcohol consumption are significant predictors of TB infection [7,23,24]. The link between alcohol use and TB could be explained by specific social mix patterns that can increase the risk of exposure to people with infectious TB disease in settings such as shelters for homeless, bars and social gatherings, as well as that alcohol may have a direct toxic effect on the immune system rendering the host more exposed to TB infection. Also, several possible mechanisms may explain the increased infectiousness of a TB patient who smokes.

For instance, according to [26], smokers cough far more frequently than nonsmokers, with individuals who are smoking cough on average 5.3 times an hour compared to 0.7 times for non-smokers. As a result, chronic coughers may be slow to recognise symptoms of a respiratory infection and therefore being late for treatment, potentially increasing the exposure of their contacts to the infection. Another possible reason may be that smoking can alter the lungs’ local immune response, promoting the continued growth of bacilli and/or the destruction of lung tissue, making it easier for a person to develop TB. As presented in these findings with regard to personal weight, underweight and normal-weight people were less likely to experience TB as compared to those who are overweight. The effect of an individual’s weight on TB transmission in the current study is overwhelming and cannot be ignored. This could be because people who are overweight have little exercise and, according to [27], promoting a healthy lifestyle will help reduce people’s risk of active TB. However, to the best of our knowledge, no study has examined the role of personal weight in the development of TB disease. Another area which most studies have not looked at is the effect of Asthma disease on TB. This study shows that people who are asthmatic are more likely to develop TB than those without asthma. This may be due to the body’s weaker immune system as it fights asthma, which allows more bacteria and viruses to enter the body and cause infections. In general, we can speculate that people with conditions that weaken the immune system are at high risk of developing TB.

In conclusion, this study has shown that age, personal weight, smoking, alcohol, asthma, province of residence, race and coughing in general are all linked to an increased risk of transmission/infection of TB after controlling for the effects of other factors. Overall, it can be said that people who have asthma and cough frequently, who are usually younger or older (not young adults), who smoke and consume alcohol are at high risk of developing TB. In addition, race and personal weight appear to be strong predictors of TB in South Africa as well, with the WC, EC, NC, FS, NW and GP being the most affected provinces. These factors must be taken into account in the planning and development of TB policies in order to work successfully towards the achievement of the sustainable development goal of reducing TB by 80% before 2030 [28,29].

## 5. Limitations, Strength and Recommendations

The results in this study should be considered with the following limitations in mind. First, the outcome measures used are self-reported data from the respondents and not laboratory-confirmed results from a doctor or a nurse. According to [30], accurate reporting is critical to controlling TB everywhere. Second, the contributing factors such as HIV status and educational level were not part of the analysis, this is certainly a major limitation of these data. HIV and education, established factors in TB found in most of the studies reviewed, could have complemented the findings of this study.

The greatest strength of this study is that it used data from a nationally representative survey and these findings can be generalised for TB in South Africa. In light of the study findings, the following recommendations are made. Firstly, secondary data were used for the study, therefore we recommend researchers who want to carry out their research to look for primary data due to the disadvantages associated with the secondary data. Secondly, future research can incorporate factors such as HIV and educational attainment into the analysis of TB transmission to determine its impact on the South African setting. Thirdly, it is necessary to conduct a similar study on risk factors in each province, mainly WC, EC, NC, FS, NW and GP in order to have a comparative analysis between the provinces. Finally, we recommend that the Ministry of Health and the various interested organizations raise awareness of the risk factors that lead to the transmission of TB in order to reduce the incidence of the disease.

## Figures and Tables

**Table 1:** Descriptive statistics for dependent and predictor variables.

Covariates	(TB%) Frequency	Percentage	Covariates	(TB%) Frequency	Percentage
Dependent variable					
**Tuberculosis**					
Yes	384	4.9%			
No	7384	95.1%			
Demographic variables			Health variables		
**Race**			**Personal Weight**		
Black/African	(6.06%) 4931	63.5%	Under Weight	(11.89%) 1320	17.0%
Coloured	(4.18%) 1700	21.9%	Normal Weight	(3.94%) 4720	60.8%
White	(1.36%) 738	9.5%	Over Weight	(2.37%) 1728	22.2%
Asian/Indian	(1.00%) 399	5.1%			
**Gender**			**Hypertension**		
Male	(5.71%) 3291	42.4%	Yes	(5.59%) 1199	15.4%
Female	(4.38%) 4477	57.6%	No	(5.84%) 6569	84.6%
**Type of place of residence**			**Asthma**		
Urban	(3.65%) 4843	62.3%	Yes	(12.31%) 333	4.3%
Rural	(7.08%) 2925	37.7%	No	(4.61%) 7435	95.7%
**Province of Residence**			**Usually Cough**		
Western Cape (WC)	(2.94%) 816	10.5%	Yes	(12.66%) 1019	13.1%
Eastern Cape (EC)	(4.62%) 2834	36.5%	No	(3.78%) 6749	86.9%
Northern Cape (NC)	(3.00%) 1167	15.0%			
Free State (FS)	(23.68%) 1111	14.3%			
KwaZul Natal (KZN)	(6.41%) 749	09.6%			
North West (NW)	(6.96%) 345	04.4%			
Gauteng (GP)	(9.94%) 533	06.9%			
Mpumalanga (MP)	(23.68%) 114	01.5%			
Limpopo (LP)	(19.19%) 99	01.3%			
**Age**					
15–24	(2.25%) 2044	26.3%			
25–34	(4.12%) 1482	19.1%			
35–44	(5.42%) 1401	18.0%			
45–54	(7.40%) 1067	13.7%			
55+	(6.88%) 1774	22.8%			
**Covariates**	**(TB%)** **Frequency**	**Percentage**	**Covariates**	**(TB%)** **Frequency**	**Percentage**
Socioeconomic variables					
**Smoke**					
Yes	(7.07%) 3164	40.7%			
No	(3.48%) 4604	59.3%			
**Alcohol**					
Yes	(6.84%) 3277	42.2%			
No	(3.56%) 4491	57.8%			
**Source of Drinking Water**					
Piped Water	(3.75%) 5177	66.6%			
Tank Water	(4.84%) 62	0.8%			
Purified Water	(7.39%) 1029	13.2%			
River Water	(6.95%) 1411	18.2%			
Borehole Water	(14.61%) 89	1.1%			
**Electricity**					
Yes	(3.58%) 5049	65.0%			
No	(7.47%) 2719	35.0%			

Keyword: TB% indicates percentage of those who had TB for different categories.

**Table 2:** Descriptive statistics for dependent and predictor variables.

Covariate	*p*-Value
Demographic variables	
Tuberculosis*Race	**0.001**
Tuberculosis*Gender	**0.007**
Tuberculosis*Province of Residence	**0.001**
Tuberculosis*Type of place of residence	0.092
Tuberculosis*Age	**0.001**
Socioeconomic explanatory variables	
Tuberculosis*Smoke	**0.001**
Tuberculosis*Alcohol	**0.001**
Tuberculosis*Source of Drinking Water	**0.001**
Tuberculosis*Electricity	**0.001**
Health variables	
Tuberculosis*Usually Cough	**0.001**
Tuberculosis*Personal Weight	**0.001**
Tuberculosis*Hypertension	0.263
Tuberculosis*Asthma	**0.001**

Keyword: * indicates crosstabulation.

**Table 3:** Multivariate analysis of factors influencing the development of tuberculosis in South Africa.

Covariate	β	SE	Wald	*p*-Value	EXP (β)	95% CI for EXP (β)
**Age**						
15–24	0.718	0.193	13.874	**0.001**	2.050	(1.405;2.990)
25–34	0.241	0.175	1.881	0.170	1.272	(0.902;1.794)
35–44	0.002	0.164	0.001	0.992	1.002	(0.726;1.382)
45–54	−0.267	0.163	2.691	**0.031**	0.762	(1.032;1.850)
55+(ref)						
**Personal Weight**						
Under Weight	−1.324	0.194	46.431	**0.001**	0.266	(0.1823;0.390)
Normal Weight	−0.564	0.184	9.426	**0.002**	0.569	(0.397;0.815)
Over Weight (ref)						
**Smoke**						
Yes	−0.303	0.138	4.776	**0.029**	0.739	(0.563;0.969)
No (ref)						
**Alcohol**						
Yes	−0.479	0.135	12.594	**0.001**	0.619	(0.475;0.807)
No (ref)						
**Asthma**						
Yes	−0.545	0.224	5.909	**0.015**	0.580	(0.374;0.900)
No (ref)						
**Province of Residence**						
Western Cape (WC)	1.742	0.370	22.143	**0.001**	5.708	(2.763;11.792)
Eastern Cape (EC)	1.919	0.300	40.999	**0.001**	6.815	(3.788;12.264)
Northern Cape (NC)	2.006	0.340	34.714	**0.001**	7.433	(3.814;14.487)
Free State (FS)	2.487	0.348	51.180	**0.001**	12.030	(6.086;23.780)
KwaZul Natal (KZN)	0.381	0.326	1.368	0.242	1.464	(0.773;2.774)
North West (NW)	1.019	0.358	8.127	**0.004**	2.771	(1.375;5.586)
Gauteng (GP)	1.567	0.336	21.814	**0.001**	4.794	(2.484;9.255)
Mpumalanga (MP)	−0.241	0.382	0.398	0.528	0.786	(0.372;1.661)
Limpopo (LP) (ref)						
**Usually Cough**						
Yes	−0.857	0.130	43.312	**0.001**	0.424	(0.329;0.548)
No(ref)						
**Race**						
Black/African	2.579	0.536	23.170	**0.001**	13.184	(1.836;6.412)
Coloured	2.181	0.559	15.220	**0.001**	8.855	(1.121;1.537)
White	−1.028	0.611	2.831	**0.092**	0.358	(0.108;1.185)
Indian (ref)						

**Table 4:** Hosmer and Lemeshow Test.

Step	Chi-Square	df	*p*-Value
8	7574	8	0.476

## Data Availability

Dataset is available on the public domain from the Statistics South Africa (StatsSA) website.
